# Recognition of acute kidney injury diagnosis in the neonatal intensive care unit

**DOI:** 10.1038/s41372-024-02095-y

**Published:** 2024-08-22

**Authors:** Katherine Vincent, Zegilor Laney, Austin Rutledge, Jill C. Newman, David T. Selewski, Heidi J. Steflik

**Affiliations:** 1 Department of Pediatrics, Medical University of South Carolina, Charleston, SC USA; 2 Department of Pediatrics, Cincinnati Children’s Medical Center, Cincinnati, OH USA; 3 Department of Medicine, Medical University of South Carolina, Charleston, SC USA

**Keywords:** Acute kidney injury, Risk factors

Acute kidney injury (AKI) occurs frequently in the neonatal intensive care unit (NICU), yet often is undetected. Multiple studies have confirmed AKI detection and documentation rates are low with only 4–26% of AKI survivors having AKI recorded in the discharge summary [1–3]. Carmody et al. found AKI occurred in ~40% of very low birthweight infants but was recorded in the discharge summary in only 13.5% of survivors [4]. The accurate diagnosis and documentation of AKI is pivotal to ensure appropriate pediatric nephrology referral occurs, a key component to ensuring subsequent follow-up of neonates at risk for chronic kidney disease (CKD) [3]. While the available literature confirms AKI is under-diagnosed in the NICU, few studies have investigated factors that may impact AKI detection.

To address this important gap, we conducted a single center, retrospective cohort study of neonates admitted to the NICU 01/01/2020–06/30/2021 to determine how frequently AKI is missed and identify factors that impact AKI recognition. Patients were excluded if they had less than 3 serum creatinine values, pre-existing end stage kidney failure, a palliative care course within the first week of life or were transferred to Medical University of South Carolina (MUSC) NICU at greater than 48 h of life. We hypothesized 40% of AKI episodes would be undiagnosed. The modified neonatal *Kidney Disease: Improving Global Outcomes* serum creatinine criteria was used to define AKI. Comparisons were made between neonates in whom AKI was identified by the medical team during the NICU stay (‘detected AKI’) and neonates in whom AKI was not diagnosed in the NICU but was identified by the research team on chart review (‘missed AKI’). Statistical methods included Chi Square, Fisher’s Exact, Student’s *t*, and Wilcoxon rank sum tests. The study was approved by the MUSC Institutional Review Board which granted a waiver of informed consent.

Of the 869 neonates included, 164 (18.9%) experienced an AKI episode while in the NICU, with a total of 308 AKI episodes. Of those neonates, 73 (44.5%) had detected AKI (174 episodes) and 91 (55.5%) had missed AKI (134 episodes). Compared to those with detected AKI, neonates with missed AKI had higher birth weight (BW, Missed: 1870 ± 888 g, Detected: 1509 ± 1124 g; *p* = 0.03), higher gestational age (GA, Missed*:* 32.4 ± 4.4 weeks, Detected: 29.0 ± 5.8 weeks; *p* < 0.01), and were more frequently male (Missed*:* 62.6%, Detected*:* 46.6%; *p* = 0.04). Neonates with missed AKI experienced shorter median durations of mechanical ventilation (Missed*:* median 2 days (0–15), Detected: 19 days (5–43); *p* < 0.01) and hospitalization (Missed: median 58 days (22–90), Detected: 75 days (28–113); *p* < 0.03), and more frequently survived (Missed 93.4%, Detected: 79.5%; *p* < 0.01).

Important differences in AKI severity were identified when comparing those with detected AKI to those with missed AKI (all *p* < 0.05, Table). The majority of missed AKI episodes were stage 1 AKI (114/134, 85.1%), but 20 episodes (14.9%) of missed AKI were stage 2 AKI (*p* < 0.05). No stage 3 AKI episodes were missed (Table 1).

**Table 1:** Comparison of acute kidney injury (AKI) episodes, risk factors, and protective factors between those with detected AKI compared to missed AKI.

AKI episode details		All neonates with AKI *n* = 164	Neonates with detected AKI *n* = 73 (44.5)	Neonates with missed AKI *n* = 91 (55.5%)	*p*-value^a^
AKI episodes		308	174 (56.5)	134 (43.5)	**NA**
Severity of AKI	KDIGO stage 1	243 (78.9)	129 (74.1)	114 (85.1)	**<0.001**
	KDIGO stage 2	52 (16.9)	32 (18.4)	20 (14.9)	
	KDIGO stage 3	13 (4.2)	13 (7.5)	0 (0)	
AKI risk and protective factors					
Patent ductus arteriosus (PDA)		93 (56.7)	57 (78.1)	36 (39.6)	**<0.001**
Sepsis		52 (31.7)	30 (41.1)	22 (24.2)	**0.021**
Nephrotoxic meds		141 (86.0)	68 (93.2)	73 (80.2)	**0.023**
Hypoxic ischemic encephalopathy (HIE)		15 (9.2)	12 (16.4)	3 (3.3)	**0.005**
Small for gestational age (SGA)		34 (20.7)	10 (13.7)	24 (26.4)	**0.047**
Intrauterine growth restriction (IUGR)		32 (19.5)	11 (15.1)	21 (23.1)	0.198
Hypotension requiring pressors		59 (36.0)	40 (54.8)	19 (20.9)	**<0.001**
Congenital heart disease		10 (6.1)	3 (4.1)	7 (7.7)	0.514
Congenital anomalies of kidney and urinary tract (CAKUT)		12 (7.3)	6 (8.2)	6 (6.6)	0.768
Caffeine exposure		86 (52.4)	47 (64.4)	39 (42.9)	**0.006**

Percentages in table are column percentages.

*n* (%), mean ± std or median [Q1, Q3].

*KDIGO* Modified neonatal Kidney Disease: Improving Global Outcomes.

The bold are statistically significant values.

^a^*p*-value from Chi Square Test, Fisher’s Exact Test, Student’s *T* test or Wilcoxon Rank Sum Test. *P*-value between neonates with detected AKI column and neonates with missed AKI column.

Known risk factors for AKI including patent ductus arteriosus, sepsis, nephrotoxic medication exposure, hypoxic ischemic encephalopathy, and hypotension requiring pressors were less common in neonates with missed AKI (all *p* < 0.05, Table 1). Caffeine citrate exposure was less common in those with missed AKI (*p* < 0.05).

Perhaps most importantly, in the cases of missed AKI, pediatric nephrology consultation was less common (Missed: 20.9%, Detected: 71.2%, *p* < 0.001) and referrals for outpatient pediatric nephrology care were less frequently completed (Missed*:* 15.4%, Detected*:* 60.3%; *p* < 0.001). However, some infants, even without AKI, were presumably referred to pediatric nephrology due to other kidney issues. Pediatric nephrology follow-up is conducted in a dedicated, pediatric nephrology clinic and consists of blood pressure measurements and serum creatinine levels.

Limitations in our study included reliance on clinical documentation in the electronic medical record which can be incomplete and the potential for missed AKI episodes because of not assessing urine output due to difficulty of measuring urine output in infants. A single neonatal AKI episode is acutely associated with increased mortality and may lead to CKD with persistence of renal dysfunction into early childhood [5]. Therefore, improvement in recognition and diagnosis of AKI remain important next steps in reducing morbidity and mortality, both in the NICU and later in life. Without early recognition, appropriate outpatient follow-up cannot occur. Following even one AKI episode, neonates are at risk of developing hypertension, hyperfiltration, proteinuria, and/or other signs of chronic kidney disease. When unrecognized these morbidities could go undetected, delaying treatment, resulting in disease progression. Collaboration between neonatology and pediatric nephrology is critical for the accurate diagnosis and documentation of AKI in critically ill neonates. Chmielewski *et al*. found pediatric nephrology consultation in neonates with AKI strongly mediated AKI diagnosis and documentation at discharge [3]. Starr et al. found standardized approaches improve neonatal AKI identification in the NICU, even when protocols vary between institutions [1]. Electronic health record-automated detection may be helpful in some centers, and ongoing education for care team members will likely be required. Further work and future studies to determine the best care model to ensure AKI recognition are needed.

Despite highly protocolized AKI care in our NICU, AKI detection remains low, particularly in neonates with higher BW, later GA, and in those who experience less severe AKI and fewer risk factors for AKI. Therefore, ongoing education is warranted, even in a setting where neonatal AKI is protocolized, researched, and frequently discussed.

## Data Availability

Currently, due to IRB restrictions, these data are not publicly available.
